# Associations between risk of Alzheimer's disease and obstructive sleep apnea, intermittent hypoxia, and arousal responses: A pilot study

**DOI:** 10.3389/fneur.2022.1038735

**Published:** 2022-11-30

**Authors:** Cheng-Yu Tsai, Sheng-Ming Wu, Yi-Chun Kuan, Yin-Tzu Lin, Chia-Rung Hsu, Wen-Hua Hsu, Yi-Shin Liu, Arnab Majumdar, Marc Stettler, Chien-Ming Yang, Kang-Yun Lee, Dean Wu, Hsin-Chien Lee, Cheng-Jung Wu, Jiunn-Horng Kang, Wen-Te Liu

**Affiliations:** ^1^Department of Civil and Environmental Engineering, Imperial College London, London, United Kingdom; ^2^Division of Pulmonary Medicine, Department of Internal Medicine, School of Medicine, College of Medicine, Taipei Medical University, Taipei, Taiwan; ^3^Division of Pulmonary Medicine, Department of Internal Medicine, Shuang Ho Hospital, Taipei Medical University, New Taipei City, Taiwan; ^4^Sleep Center, Shuang Ho Hospital, Taipei Medical University, New Taipei City, Taiwan; ^5^Department of Neurology, Shuang Ho Hospital, Taipei Medical University, New Taipei City, Taiwan; ^6^Department of Neurology, School of Medicine, College of Medicine, Taipei Medical University, Taipei, Taiwan; ^7^Taipei Neuroscience Institute, Taipei Medical University, Taipei, Taiwan; ^8^Dementia Center, Shuang Ho Hospital, Taipei Medical University, Taipei, Taiwan; ^9^Department of Medical Imaging and Intervention, Chang Gung Memorial Hospital at Linkou, Taoyuan, Taiwan; ^10^School of Respiratory Therapy, College of Medicine, Taipei Medical University, Taipei, Taiwan; ^11^Department of Psychology, National Chengchi University, Taipei, Taiwan; ^12^Department of Psychiatry, Taipei Medical University Hospital, Taipei, Taiwan; ^13^Department of Otolaryngology, Shuang Ho Hospital, Taipei Medical University, New Taipei City, Taiwan; ^14^Research Center of Artificial Intelligence in Medicine, Taipei Medical University, Taipei, Taiwan; ^15^Department of Physical Medicine and Rehabilitation, Taipei Medical University Hospital, Taipei, Taiwan; ^16^Graduate Institute of Nanomedicine and Medical Engineering, College of Biomedical Engineering, Taipei Medical University, Taipei, Taiwan

**Keywords:** obstructive sleep apnea, Alzheimer's disease, sleep-disordered breathing, total tau, amyloid beta-peptide 42, arousal response

## Abstract

**Objectives:**

Obstructive sleep apnea (OSA) may increase the risk of Alzheimer's disease (AD). However, potential associations among sleep-disordered breathing, hypoxia, and OSA-induced arousal responses should be investigated. This study determined differences in sleep parameters and investigated the relationship between such parameters and the risk of AD.

**Methods:**

Patients with suspected OSA were recruited and underwent in-lab polysomnography (PSG). Subsequently, blood samples were collected from participants. Patients' plasma levels of total tau (T-Tau) and amyloid beta-peptide 42 (Aβ_42_) were measured using an ultrasensitive immunomagnetic reduction assay. Next, the participants were categorized into low- and high-risk groups on the basis of the computed product (Aβ_42_ × T-Tau, the cutoff for AD risk). PSG parameters were analyzed and compared.

**Results:**

We included 36 patients in this study, of whom 18 and 18 were assigned to the low- and high-risk groups, respectively. The average apnea–hypopnea index (AHI), apnea, hypopnea index [during rapid eye movement (REM) and non-REM (NREM) sleep], and oxygen desaturation index (≥3%, ODI-3%) values of the high-risk group were significantly higher than those of the low-risk group. Similarly, the mean arousal index and respiratory arousal index (R-ArI) of the high-risk group were significantly higher than those of the low-risk group. Sleep-disordered breathing indices, oxygen desaturation, and arousal responses were significantly associated with an increased risk of AD. Positive associations were observed among the AHI, ODI-3%, R-ArI, and computed product.

**Conclusions:**

Recurrent sleep-disordered breathing, intermittent hypoxia, and arousal responses, including those occurring during the NREM stage, were associated with AD risk. However, a longitudinal study should be conducted to investigate the causal relationships among these factors.

## Introduction

The prevalence of obstructive sleep apnea (OSA) in the general population ranges from 9 to 38% (1). OSA is characterized by repetitive upper airway collapse, which leads to intermittent hypoxia, recurrent arousal responses, and sleep fragmentation (2). OSA is associated with a 1.26-fold risk (95% CI: 1.05–1.50) of cognitive impairment and dementia and has been linked to memory dysfunction (3, 4). One review reported that 11–71% of patients with cognitive impairment have OSA (5). In another study, more than 90% of the enrolled patients with dementia had diagnosed OSA, and 39.1% of the cases of diagnosed OSA were severe (6).

Studies have provided possible explanations of the pathological mechanisms underlying the relationship between OSA and dementia. For example, one study indicated that the respiratory events associated with OSA—namely episodes of apnea and hypopnea—affect cerebral circulation as well as cerebrovascular responses and result in hypercapnia and concomitant hypoxia (7). Intermittent hypoxia is associated with elevated reactive oxygen species formation, which can cause oxidative stress; oxidative stress can lead to inflammatory cytokine activation and, in turn, cerebral neuron impairment (8). In addition, hypercapnia and hypoxia can induce arousal responses, which are associated with fragmented sleep and an increased risk of cognitive impairment (9, 10). Therefore, exploring the effects of the various responses induced by OSA stimuli on individuals' risk of developing neurodegenerative diseases is crucial.

OSA severity is classified using the apnea–hypopnea index (AHI), which is the total number of respiratory events during sleep time. Although it can be used to assess sleep-disordered breathing, the AHI may not be entirely suitable for evaluating all phenotypic subtypes of OSA (11). For example, the AHI does not sufficiently reflect the pattern or level of sleep fragmentation or the effect of arousal responses, although these factors are correlated. Frequent arousal, which leads to sleep fragmentation, can result in neurodegeneration and is associated with dementia (12). Another study recruited male participants and investigated the relationships between brain cortical thickness and sleep parameters measured using polysomnography (PSG) among the participants with severe OSA (13). Those results indicated that the arousal index (ArI) and respiratory arousal index (R-ArI) values of the patients were significantly and negatively correlated with the cortical thickness of the prefrontal and parietal cortex areas, which may elucidate some of the underlying mechanisms of cognitive dysfunction. Additionally, researchers have established numerous animal models to investigate the association between arousal indices and biomarkers of neurodegenerative diseases. In a mouse model of Alzheimer's disease (AD), for example, increased amyloid beta peptide (Aβ) deposition was determined to be related chronic sleep fragmentation induced by intermittent nocturnal arousals (14). In other studies, sleep arousal significantly increased both Aβ and tau protein levels in the interstitial fluid of mice and reduced the clearance efficiency of these proteins in animal models (15, 16). However, the associations among sleep-disordered breathing, arousal responses, and the risk of AD development remain unclear and warrant further investigation.

This explorative study, conducted in Taiwan, investigated the associations between sleep parameters measured using PSG and plasma levels of biomarkers of neurodegenerative diseases. We also compared the sleep parameters of the patients in the low- or high-risk groups, who were grouped on the basis of biomarker levels. These aims to determine the relationships between sleep disorders and the risk of AD development. The findings of this study may help elucidate the effects of the accumulation of neurochemical biomarkers on arousal responses.

## Methods

### Ethics

The study protocol was approved by the Joint Institutional Review Board of Taipei Medical University (TMU-JIRB: N201912097), and the study was conducted in accordance with the Declaration of Helsinki. Informed consent was obtained from each participant before data collection. Participant enrollment, PSG outcome access, and blood sample collection were performed in accordance with approved guidelines.

### Study population and procedure

**Figure 1:**
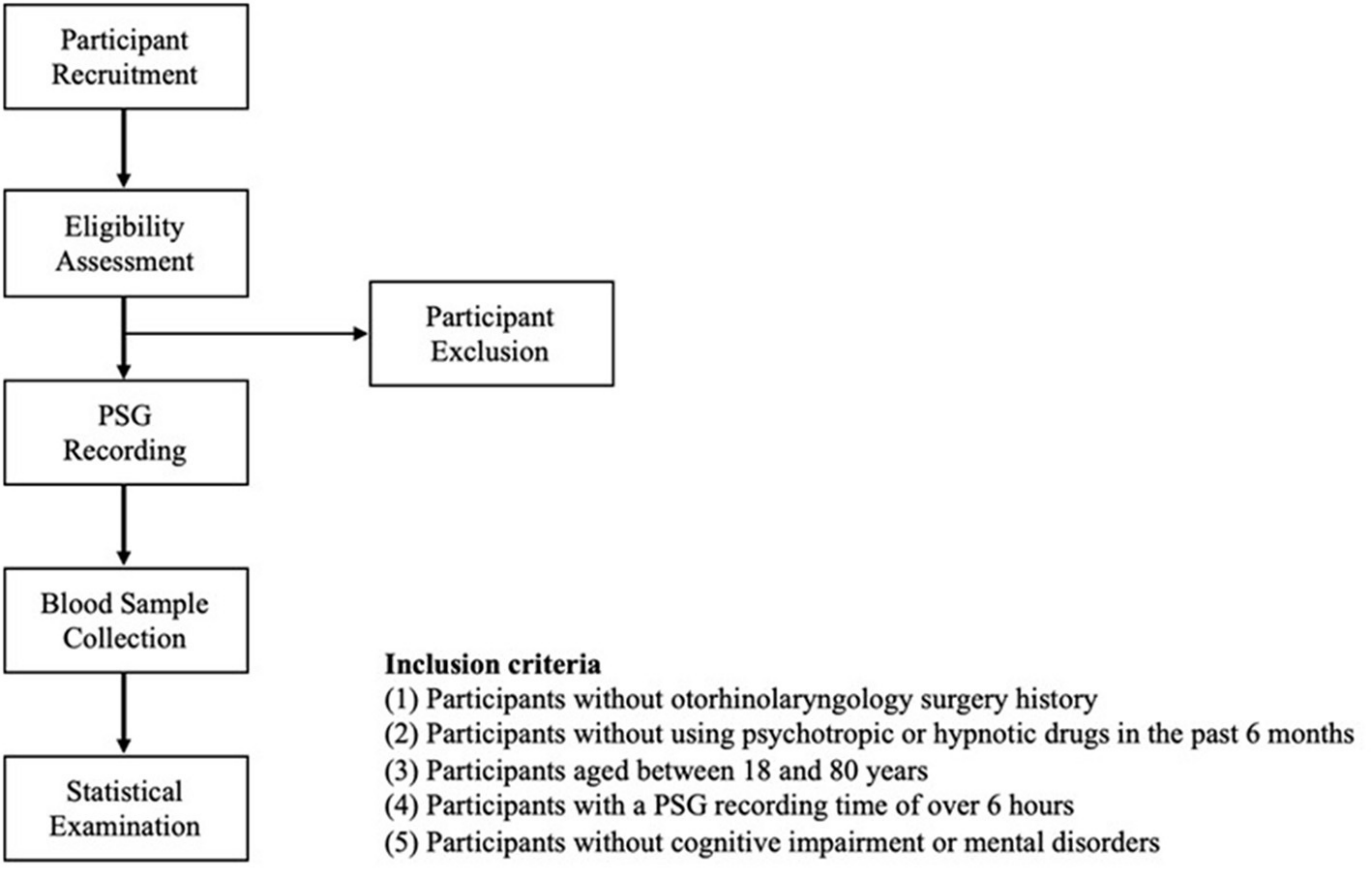
Participant recruitment flowchart. PSG, polysomnography.

Patients with suspected OSA were recruited and referred to the Sleep Center of Taipei Medical University Shuang Ho Hospital (New Taipei City, Taiwan). The participants were recruited from June to October 2018 and from July 2020 to March 2022. The recruitment criteria were as follows: (1) no history of otorhinolaryngological surgery for OSA, (2) no use of psychotropic or hypnotic drugs in the prior 6 months, (3) an age between 18 and 80 years, (4) a PSG recording of over 6 h, (5) no diagnosis of neurological disorders (e.g., AD, dementia, epilepsy, and Parkinson's disease) and other comorbidities (e.g., cardiovascular disease, renal failure, and metabolic syndrome), and (6) absence of cognitive symptoms. Figure 1 presents a flowchart of the study procedure. We arranged for the eligible participants to undergo overnight PSG at the Sleep Center. We collected blood samples from each patient in the morning at a fixed time (6:30 a.m.) after they underwent PSG to determine their plasma levels of neurochemical biomarkers. All the collected data were statistically analyzed.

### PSG

The PSG examination was performed using a ResMed Embla N7000 (ResMed, San Diego, CA, USA) and an Embla MPR (ResMed Global Supplier Alliance, Sydney, Australia) at the Sleep Center. The collected images were scored using RemLogic software (version 3.41; Embla Systems, Thornton, CO, USA) by certified PSG technologists who undergo monthly interscoring training. All the scoring rules were established in accordance with the 2017 guidelines of the American Association of Sleep Medicine (17). To ensure the consistency of the scoring, all the scoring outcomes were examined by another technologist, and any inconsistencies were resolved through discussion. Regarding the PSG parameters of interest, we collected the participants' sleep architecture–related, respiratory event–related, hypoxemia-related, and arousal-related indices. OSA severity was categorized as normal (AHI < 5 events/h), mild (AHI = 5 to 15 events/h), moderate (AHI = 15 to 30 events/h), or severe (AHI ≥ 30 events/h) (18). All arousal events were categorized as spontaneous arousal, respiratory arousal (apnea or hypopnea related), limb-movement arousal (single, periodic, or respiratory-related movement), or snore arousal on the basis of cause. The ArI value was the sum of all arousal values. In particular, abrupt alterations in electroencephalography caused by alpha (8 to 12 Hz), theta (4 to 8 Hz), or high-frequency (>16 Hz, but not with a spindle pattern) bands were scored. Alterations were recorded only if they continued for more than 3 s (≥10 s of stable sleep preceding the alterations). Next, we calculated the respiratory event–related and arousal-related indices during the rapid eye movement (REM) stage, the non-REM (NREM) stage, and total sleep time. Briefly, certified PSG technologists first examined apnea, hypopnea, oxygen desaturation (≥3%), arousal events, and sleep stages. Next, the scoring system automatically used different sleep periods to calculate the indices. The apnea index (AI) is defined as apnea events divided by total sleep time. AI_NREM_ is defined as the apnea event that occurred during the NREM period divided by NREM time. AI_REM_ is defined as the apnea event that occurred during the REM period divided by REM time. All arousal subtypes were classified on the basis of factors induced before the arousal event. For instance, respiratory arousal was defined as respiratory events, including apnea and hypopnea, that cause arousal, whereas spontaneous arousal was defined as the occurrence of arousal without the induction of any particular factor.

### Blood sample collection and processing

The blood samples of the participants were collected using a procedure described in another study (19). Approximately 16 mL of venous blood was collected from each participant and stored in a lavender-top tube coated with tripotassium ethylenediamine tetra-acetate. Within 1 h of collection, the samples were centrifuged at 2,500 *g* for 15 min at room temperature. The extracted plasma was then aliquoted into cryovials, frozen at −80°C, and delivered to MagQu (New Taipei City, Taiwan) to test the levels of neurochemical biomarkers.

### Measurement of neurochemical biomarkers in blood samples

The participants' plasma levels of total tau (T-Tau) and Aβ_42_ were determined using an ultrasensitive immunomagnetic reduction (IMR) assay. The procedure and technical details of the assay were reported in another study (20). The biomarkers Aβ_42_ and T-Tau were assayed using different reagents (MagQu, catalog number: MF-AB2-0060 and MF-TAU-0060). Mixtures of various volumes of reagents and samples were analyzed using a SQUID-based alternating-current magnetosusceptometer (model XacPro-S, MagQu, New Taipei City, Taiwan). The plasma levels of T-Tau protein and Aβ_42_ were quantified on the basis of the IMR signals generated by interaction between IMR reagents and target proteins; these signals were then converted into concentrations on the basis of the characteristic curves of each protein. The cutoff value for the computed product of Aβ_42_ and T-Tau for identifying AD (382.68 pg^2^/mL^2^, 92% accuracy in identifying AD) was established in another study (21). In this study, we used this established cutoff value to separate the recruited participants into two groups on the basis of whether they were at low or high risk of developing AD.

### Statistical analysis

All the statistical analyses were performed using SPSS version 20 (SPSS, Chicago, IL, USA). The Shapiro–Wilk test was first conducted to examine the normality of the derived parameters. Student's *t*-test and the Mann–Whitney *U* test were used to identify between-group differences in the mean values of the continuous variables with normal distributions (Shapiro–Wilk test, *p* > 0.05) and non-normal distributions (Shapiro–Wilk test, *p* < 0.05), respectively. Categorical variables were analyzed using the chi-squared test. Next, to explore the associations between the variables of the low-risk group (computed products ≤ 382.68 pg^2^/mL^2^) and those of the high-risk group (computed products > 382.68 pg^2^/mL^2^), we used simple and multivariable logistic regression models with adjustment for age, sex, and body mass index (BMI). The results are reported as crude or adjusted odds ratios (ORs) with 95% CIs. The level of significance was set to *p* < 0.05.

## Results

### Demographic characteristics of enrolled participants

Table 1 presents the demographic data of the participants. The low- and high-risk groups consisted of 18 participants each. No significant between-group differences in body-related parameters were identified. Regarding neurochemical biomarkers levels, compared with the low-risk group, the high-risk group had significantly higher levels of T-Tau and Aβ_42_ as well as a higher average ratio (Aβ_42_/T-Tau) and product (Aβ_42_ × T-Tau) of the two. In addition, the OSA severity distributions of the groups did not differ significantly (*p* = 0.11); most of the participants in both groups had severe OSA (low-risk group: 61.11%; high-risk group: 88.89%).

**Table 1:** Comparison of demographic characteristics of the low- and high-risk groups.

**Categorical variable**	**Low-risk group** ** (*n* = 18)**	**High-risk group** ** (*n* = 18)**	***p***
Age (y)^a^	51.72 ± 11.4	52.78 ± 11.24	0.90
Sex (male/female)^b^	10/8	13/5	0.30
BMI (kg/m^2^)^a^	29.5 ± 5.22	28.62 ± 3.82	0.62
Neck circumference (cm)^a^	41.67 ± 10.44	38.56 ± 3.05	0.60
Waist circumference (cm)^a^	95.36 ± 20.19	96.72 ± 9.76	0.99
**Biomarker levels** ^**a**^			
T-Tau (pg/mL)	20.31 ± 2.62	27.39 ± 4.57	< 0.01
Aβ_42_ (pg/mL)	15.83 ± 0.72	16.98 ± 0.85	< 0.01
Aβ_42_/T-Tau	0.79 ± 0.1	0.63 ± 0.08	< 0.01
Aβ_42_ × T-Tau (pg/mL)^2^	322.02 ± 46.75	467.79 ± 102.24	< 0.01
**OSA severity** ^**b**^			0.11
Normal, *n* (%)	1 (5.56%)	–	
Mild, *n* (%)	4 (22.22%)	–	
Moderate, *n* (%)	2 (11.11%)	2 (11.11%)	
Severe, *n* (%)	11 (61.11%)	16 (88.89%)	

BMI, body mass index; OSA, obstructive sleep apnea.

Data are expressed as means ± standard deviations.

a Differences between groups were assessed using Mann–Whitney U test.

b Differences between groups were assessed using the chi-squared test.

### Sleep parameters

Table 2 presents the participants' sleep architecture parameters, oximetry parameters, and sleep-disordered breathing indices. Regarding peripheral arterial oxygen saturation (SpO_2_) indices, compared with the low-risk group, the high-risk group had significantly higher oxygen desaturation index values (≥3%, ODI-3%; *p* < 0.01) and lower minimum SpO_2_ (*p* < 0.05). Regarding sleep-disordered breathing indices, the low-risk group had significantly lower AHI, AI, and hypopnea index (HI) values than did the high-risk group (AHI: 28.55 ± 15.56 vs. 52.59 ± 17.28 events/h, *p* < 0.01; AI: 6.33 ± 8.03 vs. 19.53 ± 16.4 events/h*, p* < 0.01; HI: 22.22 ± 13.23 vs. 33.07 ± 14.39 events/h*, p* < 0.05). Regarding the indices calculated during NREM sleep, the high-risk group had significantly higher mean AI_NREM_ and HI_NREM_ values. No significant between-group difference in sleep-disordered breathing indices calculated during REM sleep was identified.

**Table 2:** Comparison of sleep parameters of the low- and high-risk groups.

**Categorical variable**	**Low-risk group** ** (*n* = 18)**	**High-risk group** ** (*n* = 18)**	***p***
**Sleep architecture parameters**			
Sleep efficiency (%)	79.16 ± 15.53	77.55 ± 13.37	0.39
Wake (% of SPT)	17.46 ± 14.49	17.08 ± 11.16	0.75
NREM (% of SPT)	71.97 ± 11.57	70.32 ± 10.41	0.67
REM (% of SPT)	10.55 ± 6.81	12.6 ± 5.52	0.33
WASO (min)	60.04 ± 48.7	57.65 ± 32.42	0.82
TST (min)	288.97 ± 57.25	285.21 ± 49.4	0.54
**Oximetry parameters**			
Mean SpO_2_ (%)	95.19 ± 1.63	93.98 ± 2.1	0.06
Minimum SpO_2_ (%)	83.17 ± 7.65	76.39 ± 8.61	< 0.05
ODI-3% (events/h)	25.16 ± 15.11	47.16 ± 16.56	< 0.01
**SDB indices (events/h)**			
AHI	28.55 ± 15.56	52.59 ± 17.28	< 0.01
AI	6.33 ± 8.03	19.53 ± 16.4	< 0.01
AI_NREM_	5.38 ± 8.76	19.39 ± 17.5	< 0.01
AI_REM_	12.26 ± 15.45	21.34 ± 18.01	0.13
HI	22.22 ± 13.23	33.07 ± 14.39	< 0.05
HI_NREM_	21.2 ± 13.3	33.38 ± 14.66	< 0.05
HI_REM_	27.24 ± 21.18	33.05 ± 20.47	0.41

SPT, Sleep period time; NREM, non-rapid eye movement; REM, rapid eye movement; WASO, wake time after sleep onset; TST, total sleep time; SpO_2_, peripheral arterial oxygen saturation (as measured using pulse oximetry); ODI-3%, oxygen desaturation index ≥3%; SDB, sleep-disordered breathing; AHI, apnea–hypopnea index; AI, apnea index; AI_*NREM*_, apnea index in non-rapid eye movement stage; AI_*REM*_, apnea index in rapid eye movement stage; HI, hypopnea index; HI_*NREM*_, hypopnea index in non-rapid eye movement stage; HI_*REM*_, hypopnea index in rapid eye movement stage.

Data are expressed as means ± standard deviations.

Differences between groups were assessed using the Mann–Whitney U test.

### Arousal parameters

Table 3 presents the participants' arousal-related parameters during the REM and NREM stages and total sleep time. The ArI and R-ArI of the low-risk group were significantly lower than those of the high-risk group (ArI: 17.44 ± 8.94 vs. 28.46 ± 15.95 events/h*, p* < 0.05; R-ArI: 8.87 ± 6.44 vs. 20.93 ± 14.93 events/h*, p* < 0.01). Regarding NREM sleep parameters, the ArI_NREM_ and R-ArI_NREM_ of the high-risk group were significantly higher than those of the low-risk group (ArI_NREM_: 16.59 ± 8.35 vs. 28.91 ± 16.97 events/h*, p* < 0.05; R-ArI_NREM_: 8.05 ± 6.14 vs. 21.2 ± 15.95 events/h*, p* < 0.01). In addition, the high-risk group had a significantly higher mean R-ArI_REM_ than did the low-risk group (*p* < 0.05). However, no significant between-group differences in the types of spontaneous arousal were observed.

**Table 3:** Comparison of arousal indices of the low- and high-risk groups.

**Variable (events/h)**	**Low-risk group** ** (*n* = 18)**	**High-risk group** ** (*n* = 18)**	***p***
ArI	17.44 ± 8.94	28.46 ± 15.95	< 0.05
ArI_NREM_	16.59 ± 8.35	28.91 ± 16.97	< 0.05
ArI_REM_	23.54 ± 17.3	25.36 ± 13.55	0.73
Sp-ArI	6.61 ± 4.45	5.51 ± 5.01	0.27
Sp-ArI_NREM_	6.64 ± 4.48	5.62 ± 5.12	0.25
Sp-ArI_REM_	9.09 ± 9.17	4.31 ± 5.78	0.05
R-ArI	8.87 ± 6.44	20.93 ± 14.93	< 0.01
R-ArI_NREM_	8.05 ± 6.14	21.2 ± 15.95	< 0.01
R-ArI_REM_	12.44 ± 15.49	19.12 ± 13.93	< 0.05
Sn-ArI	0.26 ± 1.11	0.45 ± 1.32	0.62
Sn-ArI_NREM_	0.26 ± 1.08	0.47 ± 1.39	0.57
Sn-ArI_EM_	0.31 ± 1.3	0.34 ± 1.0	0.62
L-ArI	1.64 ± 2.05	1.63 ± 2.14	0.79
L-ArI_NREM_	1.69 ± 3.7	1.55 ± 2.93	0.71
L-ArI_REM_	1.72 ± 2.03	1.58 ± 2.09	0.63

ArI, arousal index; ArI_*NREM*_, arousal index in non-rapid eye movement stage; ArI_*REM*_, arousal index in rapid eye movement stage; Sp-ArI, spontaneous arousal index; Sp-ArI_*NREM*_, spontaneous arousal index in non-rapid eye movement stage; Sp-ArI_*REM*_, spontaneous arousal index in rapid eye movement stage; R-ArI, respiratory arousal index; R-ArI_*NREM*_, respiratory arousal index in non-rapid eye movement stage; R-ArI_*REM*_, respiratory arousal index in rapid eye movement stage; Sn-ArI, snore arousal index; Sn-ArI_*NREM*_, snore arousal index in non-rapid eye movement stage; Sn-ArI_*REM*_, snore arousal index in rapid eye movement stage; L-ArI, limb movement arousal index; L-ArI_*NREM*_, limb movement arousal index in non-rapid eye movement stage; L-ArI_*REM*_, limb movement arousal index in rapid eye movement stage.

Data are expressed as means ± standard deviations.

Differences between groups were assessed using the Mann–Whitney U test.

### Elevated sleep-disordered breathing indices are associated with a higher risk of AD development

Table 4 presents the associations among the sleep-disordered breathing indices of the low- and high-risk groups determined using the logistic regression models. An increase of 1 event of ODI-3% per hour was associated with a 1.10-fold higher OR (95% CI: 1.03–1.17, *p* < 0.01) and a 1.13-fold higher OR (95% CI: 1.05–1.21, *p* < 0.01) for developing AD in the crude model and the model adjusted for age, sex, and BMI. Similarly, we determined the statistically significant ORs for developing AD for each event-per-hour increase in the following parameters: AHI (crude OR 1.10, 95% CI 1.03–1.18, *p* < 0.01; adjusted OR 1.13, 95% CI 1.05–1.18, *p* < 0.01), AI (crude OR 1.10, 95% CI 1.02–1.20, *p* < 0.05; adjusted OR 1.10, 95% CI 1.01–1.2*, p* < 0.05), and HI (crude OR 1.06, 95% CI 1.0–1.12, *p* < 0.05; adjusted OR 1.12, 95% CI 1.02–1.22, *p* < 0.05). Similar results were obtained when the analysis was restricted to parameters measured during NREM sleep (AI_NREM_: crude OR 1.09, 95% CI 1.01–1.18, *p* < 0.05; adjusted OR 1.09, 95% CI 1.01–1.2, *p* < 0.05; HI_NREM_: Crude OR 1.07, 95% CI 1.01–1.13, *p* < 0.05; adjusted OR 1.12, 95% CI 1.02–1.22, *p* < 0.05).

**Table 4:** Odd ratios (ORs) associated with the sleep-disordered breathing indices of the low- and high-risk groups.

**Variable (events/h)**	**Crude OR**	**Adjusted OR**
	**(95% CI)^a^**	**(95% CI)^b^**
Oximetry parameter		
ODI-3% (events/h)	1.10 (1.03–1.17)^**^	1.13 (1.05–1.21)^**^
SDB index (events/h)		
AHI	1.10 (1.03–1.18)^**^	1.13 (1.05–1.21)^**^
AI	1.06 (1.01–1.10)^*^	1.10 (1.01–1.2)^*^
AI_NREM_	1.09 (1.01–1.18)^*^	1.09 (1.01–1.18)^*^
AI_REM_	1.03 (0.99–1.08)	1.04 (0.99–1.09)
HI	1.06 (1.0–1.12)^*^	1.12 (1.02–1.22)^*^
HI_NREM_	1.07 (1.01–1.13)^*^	1.12 (1.02–1.22)^*^
HI_REM_	1.01 (0.98–1.05)	1.03 (0.98–1.08)

ODI-3%, oxygen desaturation index ≥3%; SDB, Sleep-disordered breathing; AHI, apnea–hypopnea index; AI, apnea index; AI_*NREM*_, apnea index in non-rapid eye movement stage; AI_*REM*_, apnea index in rapid eye movement stage; HI, hypopnea index; HI_*NREM*_, hypopnea index in non-rapid eye movement stage; HI_*REM*_, hypopnea index in rapid eye movement stage.

a Simple logistic regression models.

b Multivariable logistic regression models adjusted for age, sex, and body mass index.

* p < 0.05;

** p < 0.01.

### Elevated arousal indices are associated with a higher risk of AD development

Table 5 presents the associations among the arousal indices of the low- and high-risk groups determined using the logistic regression models. Every event-per-hour increase in ArI and R-ArI was associated with a 1.08-fold higher OR (95% CI: 1.01–1.15, *p* < 0.05) and 1.16-fold higher OR (95% CI: 1.03–1.31, *p* < 0.05) for developing AD, respectively. After adjustment for age, sex, and BMI, each event-per-hour increase in ArI and R-ArI was significantly associated with a 1.08-fold higher OR (95% CI: 1.0–1.16, *p* < 0.05) and 1.16-fold higher OR (95% CI: 1.02–1.31, *p* < 0.05) of developing AD, respectively. When the analysis was restricted to arousal indices measured during NREM sleep, we identified similar trends in ArI_NREM_ and R-ArI_NREM_ (ArI_NREM_: crude OR 1.08, 95% CI 1.01–1.16, *p* < 0.05; adjusted OR 1.08, 95% CI 1.01–1.16, *p* < 0.05; R-ArI_NREM_: crude OR 1.15, 95% CI 1.03–1.3, *p* < 0.05; adjusted OR 1.15, 95% CI 1.02–1.29, *p* < 0.05).

**Table 5:** Odd ratios associated with arousal indices of the low- and high-risk groups.

**Variable (events/h)**	**Crude OR**	**Adjusted OR**
	**(95% CI)^a^**	**(95% CI)^b^**
ArI	1.08 (1.01–1.15)^*^	1.08 (1.0–1.16)^*^
ArI_NREM_	1.08 (1.01–1.16)^*^	1.08 (1.01– 1.16)^*^
ArI_REM_	1.01 (0.97–1.05)	1.0 (0.96–1.05)
Sp-ArI	0.95 (0.82–1.1)	0.98 (0.84–1.14)
Sp-ArI_NREM_	0.95 (0.83–1.1)	0.99 (0.85– 1.15)
Sp-ArI_REM_	0.92 (0.83–1.01)	0.92 (0.84–1.02)
R-ArI	1.16 (1.03–1.31)^*^	1.16 (1.02–1.31)^*^
R-ArI_NREM_	1.15 (1.03–1.3)^*^	1.15 (1.02– 1.29)^*^
R-ArI_REM_	1.03 (0.98–1.08)	1.03 (0.98–1.09)

CI, confidence interval; ArI, arousal index; ArI_*NREM*_, arousal index in non-rapid eye movement stage; ArI_*REM*_, arousal index in rapid eye movement stage; Sp-ArI, spontaneous arousal index; Sp-ArI_*NREM*_, spontaneous arousal index in non-rapid eye movement stage; Sp-ArI_*REM*_, spontaneous arousal index in rapid eye movement stage; R-ArI, respiratory arousal index; R-ArI_*NREM*_, respiratory arousal index in non-rapid eye movement stage; R-ArI_*REM*_, respiratory arousal index in rapid eye movement stage.

a Simple logistic regression models.

b Multivariable logistic regression models adjusted for age, sex, and body mass index.

* p < 0.05.

### Associations between sleep parameters and computed product (Aβ_42_ × T-Tau)

Table 6 presents a summary of the associations between the computed product and sleep parameters, including oximetry, sleep-disordered breathing, and arousal, determined using multivariable linear regression after adjustment for age, sex, and BMI. Each additional ODI-3% and AHI event occurring per hour of sleep was significantly associated with an elevated level of the computed product [ODI-3%: 0.41, 95% confident interval (CI): 0.08 to 0.74, *P* < 0.05; AHI: 0.40, 95% CI: 0.06–0.74, *P* < 0.05]. For the arousal index, every event-per-hour increase in R-ArI and R-ArI_NREM_ was significantly associated with an increased level of the computed product (R-ArI: 0.34, 95% CI: 0.02–0.66, *P* < 0.05; AHI: 0.35, 95% CI: 0.04–0.67, *P* < 0.05).

**Table 6:** Associations between sleep parameters and computed product (Aβ_42_ × T-Tau).

**Variable (events/h)**	**Beta coefficient**
	**(95% confidence interval)**
Oximetry parameters	
ODI-3% (events/h)	0.41 (0.08 to 0.74)^*^
SDB indices (events/h)	
AHI	0.40 (0.06 to 0.74)^*^
AI	0.21 (−0.12 to 0.54)
AI_NREM_	0.20 (−0.13 to 0.54)
AI_REM_	0.19 (−0.16 to 0.54)
HI	0.35 (−0.03 to 0.72)
HI_NREM_	0.36 (−0.0 to 0.72)
HI_REM_	0.17 (−0.25 to 0.59)
Arousal indices (events/h)
ArI	0.26 (−0.07 to 0.59)
ArI_NREM_	0.26 (−0.06 to 0.59)
ArI_REM_	0.08 (−0.3 to 0.45)
Sp-ArI	−0.11 (−0.46 to 0.24)
Sp-ArI_NREM_	−0.12 (−0.47 to 0.23)
Sp-ArI_REM_	−0.12 (−0.46 to 0.21)
R-ArI	0.34 (0.02 to 0.66)^*^
R-ArI_NREM_	0.35 (0.04 to 0.67)^*^
R-ArI_REM_	0.13 (−0.24 to 0.51)

ODI-3%, oxygen desaturation index ≥3%; SDB, sleep-disordered breathing; AHI, apnea–hypopnea index; AI, apnea index; AI_*NREM*_, apnea index in the non-rapid eye movement stage; AI_*REM*_, apnea index in rapid eye movement stage; HI, hypopnea index; HI_*NREM*_, hypopnea index in non-rapid eye movement stage; HI_*REM*_, hypopnea index in rapid eye movement stage. ArI, arousal index; ArI_*NREM*_, arousal index in non-rapid eye movement stage; ArI_*REM*_, arousal index in rapid eye movement stage; Sp-ArI, spontaneous arousal index; Sp-ArI_*NREM*_, spontaneous arousal index in non-rapid eye movement stage; Sp-ArI_*REM*_, spontaneous arousal index in rapid eye movement stage; R-ArI, respiratory arousal index; R-ArI_*NREM*_, respiratory arousal index in non-rapid eye movement stage; R-ArI_*REM*_, respiratory arousal index in rapid eye movement stage.

Multivariable linear regression models were adjusted for age, sex, and body mass index.

* p < 0.05.

### Determination of odds ratio by using different cutoff points

To enhance the robustness of the observed outcomes, we employed another cutoff for the product (Aβ42 × T-Tau; 403.72 pg^2^/mL^2^), which was determined using the IMR technique (22). According to this cutoff, 14 and 22 patients were included in the high- and low-risk groups, respectively. Supplementary Tables S2, S3 present associations among the sleep-disordered breathing indices and arousal indices, respectively, in the low- and high-risk groups determined using logistic regression models. Significant associations among ODI-3%, AHI, HI, and HI_NREM_ were observed in both crude and adjusted models (adjusted for age, sex, and BMI). In terms of the arousal effect, R-ArI was significantly associated with the risk of AD in both crude and adjusted models. When the analysis was restricted to only the NREM period, significant associations were observed between ArI_NREM_ and R-ArI_NREM_ in both crude and adjusted models.

## Discussion

Although OSA has been determined to be associated with the formation and accumulation of neurochemical biomarkers, the relationship between the clinical symptoms of OSA, such as intermittent hypoxia and arousal responses, and levels of neurochemical biomarker has not been explored. Therefore, we compared the sleep parameters (measured using PSG) of individuals at low and high risks of developing AD and explored the associations between the participants' sleep parameters and neurochemical biomarker levels. The results reveal that the high-risk group had significantly higher mean values for various indices of sleep-disordered breathing and arousal responses than the low-risk group. We determined that increased risk of developing AD was associated with various arousal response and sleep-disordered breathing indices.

The mean AI, HI, AI_NREM_, HI_NREM_, ODI-3%, and AHI of the low- and high-risk groups differed significantly despite the lack of significant differences between the OSA severity distributions of the groups. Moreover, significant and positive associations were observed among the AHI, ODI-3%, and computed product. These findings are consistent with those of studies investigating the association between oxygen desaturation and elevated levels of neurochemical biomarkers (23, 24) that serve as indicators of hypoxia, a major risk factor for neurochemical biomarker accumulation. Hypoxia causes neuronal apoptosis and tau hyperphosphorylation (25). One study reported a significant association between high AHI values and neurochemical biomarker levels in American patients with severe OSA without dementia (26). Another prospective study analyzed the sleep disorder characteristics of 298 older women (aged ≥ 65 years) without dementia and reported that an increased oxygen desaturation index (≥15 events/h) was associated with the risk of mild cognitive impairment or dementia after adjustment for age, BMI, and ethnicity (27). Hypercapnia, another key risk factor of OSA, can cause deterioration of the functional and anatomic status of cerebral vessels, which may lead to AD (28). Taken together, the available evidence suggests that sleep-disordered breathing is related to an individual's risk of developing AD.

Regarding arousal responses, the patients in the high-risk group had significantly higher ArI, R-ArI, ArI_NREM_, and R-ArI_NREM_ values than did those in the low-risk group. Moreover, R-ArI and R-ArI_NREM_ values were positively associated with the increased computed product. These results may be attributed to the pathogenic mechanisms of sleep arousal. Specifically, arousal responses refer to abrupt alterations between sleep and fractional wakefulness (29). Recurrent sleep arousal can interrupt the sleep cycle, alter the sleep architecture, and affect the metabolism of neurodegenerative biomarkers (30). They may also be attributed to the tendency of arousal to disrupt the clearance of neurotoxic proteins, resulting in the increased formation of amyloid plaques and the hyperphosphorylation of tau protein strands in the brain, as demonstrated in another study (31). One review similarly concluded that sleep fragmentation and nighttime awakening were associated with AD progression (32). In the present study, the groups' mean arousal indices measured during NREM sleep differed significantly. This may be explained by the underlying mechanisms of slow-wave sleep, which only occurs in the NREM stage and is associated with the modulation of neurochemical biomarkers; that is, arousals during NREM sleep are likely to interfere with the clearance of neurotoxic proteins. Studies have indicated that lessened or unstable NREM sleep increases the level of neurochemical biomarkers, which is consistent with the findings of the present study (33, 34). Collectively, the results of the present study indicate that the high-risk group had higher mean values for the selected arousal indices because arousal responses may disrupt the clearance of neurotoxic proteins, resulting in sleep fragmentation and, in turn, an increased risk of developing AD.

We further explored the relationships between sleep parameters and the risk of AD development by using logistic regression models with and without adjustment for demographic characteristics. Our findings indicate that frequent respiratory events, intermittent hypoxic episodes, and respiratory arousal responses were significantly associated with an increased risk of developing AD. These findings may be attributable to various risk factors, including hypoxia, oxidative stress, and sleep cycle fragmentation. One review elucidated the pathological roles of hypoxia in AD, which include facilitating the accumulation of neurotoxic proteins, enhancing the hyperphosphorylation of tau protein, diminishing the function of the blood–brain barrier, and accelerating neurodegeneration (35). Research has demonstrated the relationship among AD pathology, oxidative stress, and oxidative damage to the brain (36). Another study reported that sleep discontinuity interfered with the clearance of neurotoxic proteins from the central nervous system by the glymphatic system (37). Similarly, another study suggested that sleep disturbance may cause systemic inflammation, thereby increasing Aβ accumulation, which is thought to be a driver of AD pathogenesis (38). Taken together, the findings of this study reveal that high indices of sleep-disordered breathing and hypoxia and a high frequency of respiratory arousal are associated with an increased risk of developing AD.

The present study has some strengths. First, this study analyzed data derived from patients without cognitive symptoms and observed positive associations among sleep-disordered breathing events, neuron biomarker levels, and AD risk. These outcomes are in accordance with the findings of several studies indicating an association of OSA with AD risk (23). Moreover, in contrast to previous studies analyzing sleep parameters in patients with AD (6, 39), this pilot study focused on patients without any cognitive impairment symptom, and the findings of this study may help in understanding the relationship between OSA and AD risk. Another major finding of this pilot study is the positive associations between the respiratory arousal frequency and neuron biomarker levels. Previous studies have investigated the associations between arousal responses and cognitive impairment or different neuron biomarker plasma levels in children (40, 41). The results of this study demonstrated that respiratory arousal events were associated with elevated neuron biomarker levels and thus increased AD risk in adult patients without cognitive impairment. The findings of this pilot study suggest that sleep-disordered breathing and related arousal responses may increase neuron biomarker levels and thus AD risk.

This study has several limitations that should be addressed. First, although individuals with diagnosed AD were excluded from this study, we did not perform neuropsychological evaluations to assess the brain function of the enrolled participants. Moreover, we did not enroll patients who had cognitive symptoms or were diagnosed as having neurological disorders. The mean age of the enrolled patients was 52.25 years; thus, they may have a relatively low risk of cognitive impairment (42). Nevertheless, future studies should investigate the associations among PSG parameters, cognitive questionnaire responses, and biomarker levels to enhance the robustness of our results. Next, during the recruitment process, the presence of genes associated with neurodegenerative diseases was not determined to be predictive of an individual's risk of developing neurodegenerative diseases. However, the potential effects of genetic factors (e.g., *ApoE4*) on the participants' baseline levels of neurochemical biomarkers and the risk of AD development must be taken into account (43). We did not measure the levels of biomarkers in the participants' cerebrospinal fluid to compare against their levels in the plasma samples, and only one cross-sectional measurement was performed. We were therefore unable to track shifts in biomarker levels over time or evaluate the potential causal relationship between sleep parameters and individuals' risk of AD development. Next, this study did not measure other biomarkers in plasma, such as p-tau 181, Aβ_40_, or neurofilament light chain protein. These related biomarkers may help identify the risk of AD and thus the associations between OSA and AD. Although we observed positive associations between sleep-disordered breathing indices and the computed product (Aβ_42_ × T-Tau), we did not measure the levels of inflammatory biomarkers (i.e., glial fibrillary acidic protein). Examination of the level of this biomarker can help in identifying the relationships among hypoxia, inflammation, and neuron impairment. These limitations should be addressed in long-term follow-up studies involving cohorts of patients with OSA.

Another limitation of this study is that it included a small sample of participants enrolled from a single sleep center. Such a small sample size may affect the generalization of our results to different populations. In addition, we recruited individuals only from a single region in Taiwan as the study population. The relationships between arousal indices and neurochemical biomarkers should be further explored in multicenter studies. Previous studies have indicated that the ratio of slow oscillations in the N3 stage was linked to cognitive impairment or AD risk (44). However, this study calculated only the sleep index during the NREM period to eliminate the first-night effect of PSG instead of splitting them into N1, N2, and N3 stages. The first-night effect, resulting from the sleep laboratory environment or PSG devices, may reduce slow-wave sleep and thus cause the incorrect estimation of sleep parameters in some particular sleep stages (e.g., sleep indices in the N3 stage may be overestimated due to the short N3 period) (45). However, respiratory and arousal events occurring in the N3 stage may be crucial risk factors interrupting neuron biomarker clearance. Therefore, researchers should include a large sample and individuals of different ethnicities as well as perform multiple-night PSG to increase the number of participants and investigate sleep parameters in each sleep stage but with the elimination of the first-night effect.

## Conclusion

In this study, drawing on the PSG data and plasma levels of selected biomarkers of a sample population from northern Taiwan, we observed that the group that was at high risk of developing AD (patients with computed products > 382.68 pg^2^/mL^2^) had higher mean values for several sleep-disordered breathing and arousal indices (during both the REM and NREM stages) than did the group that was at low risk of developing AD. In addition, higher values for sleep-disordered breathing indices, namely AI, HI, AI_NREM_, HI_NREM_, AHI, and ODI-3%, were associated with an increased risk of AD. Arousal responses, especially respiratory arousal responses, were also associated with an increased risk of AD. Moreover, sleep-disordered breathing indices (AHI and ODI-3%) and respiratory arousal indices (R-ArI and R-ArI_NREM_) were positively associated with the computed product. These results indicate that respiratory events, intermittent hypoxia, and arousal responses, including those that occur during the NREM stage, are associated with an increased risk of developing AD. However, the causal relationships among these factors must be further explored through a longitudinal study.

## Data availability statement

The raw data supporting the conclusions of this article will be made available by the corresponding author without undue reservation.

## Ethics statement

The studies involving human participants were reviewed and approved by the Joint Institutional Review Board of Taipei Medical University TMU-JIRB: N201912097. The patients/participants provided their written informed consent to participate in this study.

## Author contributions

Conceptualized and designed the study: C-YT, S-MW, and W-TL. Data curation and investigation: Y-CK, C-RH, W-HH, Y-SL, and C-MY. Analyzed the data and drafted the paper: C-YT, S-MW, Y-CK, Y-TL, and MS. Critically revised the manuscript and provided essential intellectual contributions: W-TL, AM, C-MY, DW, H-CL, and C-JW. Project administration: K-YL, J-HK, and W-TL. All authors have approved the final version of the manuscript for publication.

## Funding

This study was supported by the Ministry of Science and Technology of Taiwan (Grant Number: MOST 111-2634-F-002 -021-) and Taipei Medical University Shuang Ho Hospital (Grant Number: 108TMU-SHH-08). The funders had no role in the study design, data collection and analysis, decision to publish, or preparation of the manuscript.

## Conflict of interest

The authors declare that the research was conducted in the absence of any commercial or financial relationships that could be construed as a potential conflict of interest.

## Publisher's note

All claims expressed in this article are solely those of the authors and do not necessarily represent those of their affiliated organizations, or those of the publisher, the editors and the reviewers. Any product that may be evaluated in this article, or claim that may be made by its manufacturer, is not guaranteed or endorsed by the publisher.

## References

[1] Senaratna CV Perret JL Lodge CJ Lowe AJ Campbell BE Matheson MC. Prevalence of obstructive sleep apnea in the general population: a systematic review. Sleep Med Rev. (2017) 34:70–81. 10.1016/j.smrv.2016.07.002 27568340

[2] Lévy P Ryan S Oldenburg O Parati G. Sleep apnoea and the heart. Eur Respir Rev. (2013) 22:333–52. 10.1183/09059180.00004513 23997061 PMC9487359

[3] Leng Y McEvoy CT Allen IE Yaffe K. Association of sleep-disordered breathing with cognitive function and risk of cognitive impairment: a systematic review and meta-analysis. JAMA Neurol. (2017) 74:1237–45. 10.1001/jamaneurol.2017.2180 28846764 PMC5710301

[4] Shastri A Bangar S Holmes J. Obstructive sleep apnoea and dementia: is there a link? Int J Geriatr Psychiatry. (2016) 31:400–5. 10.1002/gps.4345 26266479

[5] Mubashir T Abrahamyan L Niazi A Piyasena D Arif AA Wong J. The prevalence of obstructive sleep apnea in mild cognitive impairment: a systematic review. BMC Neurol. (2019) 19:195. 10.1186/s12883-019-1422-3 31416438 PMC6694482

[6] Gaeta AM Benítez ID Jorge C Torres G Dakterzada F Minguez O. Prevalence of obstructive sleep apnea in Alzheimer's disease patients. J Neurol. (2020) 267:1012–22. 10.1007/s00415-019-09668-4 31832828

[7] Durgan DJ Bryan RM Jr. Cerebrovascular consequences of obstructive sleep apnea. J Am Heart Assoc. (2012) 1:e000091. 10.1161/JAHA.111.000091 23130152 PMC3487354

[8] Zhou L Chen P Peng Y Ouyang R. Role of oxidative stress in the neurocognitive dysfunction of obstructive sleep apnea syndrome. Oxid Med Cell Longevity. (2016) (2016) 2016:9626831. 10.1155/2016/9626831 27774119 PMC5059616

[9] Gagnon K Baril A-A Gagnon J-F Fortin M Décary A Lafond C. Cognitive impairment in obstructive sleep apnea. Pathol Biol. (2014) 62:233–40. 10.1016/j.patbio.2014.05.015 25070768

[10] Souza GM Stornetta RL Stornetta DS Abbott SB Guyenet PG. Contribution of the retrotrapezoid nucleus and carotid bodies to hypercapnia-and hypoxia-induced arousal from sleep. J Neurosci. (2019) 39:9725–37. 10.1523/JNEUROSCI.1268-19.2019 31641048 PMC6891059

[11] Zinchuk A Yaggi HK. Phenotypic subtypes of OSA: a challenge and opportunity for precision medicine. Chest. (2020) 157:403–20. 10.1016/j.chest.2019.09.002 31539538 PMC7005379

[12] Lim AS Kowgier M Yu L Buchman AS Bennett DA. Sleep fragmentation and the risk of incident Alzheimer's disease and cognitive decline in older persons. Sleep. (2013) 36:1027–32. 10.5665/sleep.2802 23814339 PMC3669060

[13] Joo EY Jeon S Kim ST Lee J-M Hong SB. Localized cortical thinning in patients with obstructive sleep apnea syndrome. Sleep. (2013) 36:1153–62. 10.5665/sleep.2876 23904675 PMC3700712

[14] Minakawa EN Miyazaki K Maruo K Yagihara H Fujita H Wada K. Chronic sleep fragmentation exacerbates amyloid β deposition in Alzheimer's disease model mice. Neurosci Lett. (2017) 653:362–9. 10.1016/j.neulet.2017.05.054 28554860

[15] Holth JK Fritschi SK Wang C Pedersen NP Cirrito JR Mahan TE. The sleep-wake cycle regulates brain interstitial fluid tau in mice and CSF tau in humans. Science. (2019) 363:880–4. 10.1126/science.aav2546 30679382 PMC6410369

[16] Wang C Holtzman DM. Bidirectional relationship between sleep and Alzheimer's disease: role of amyloid, tau, and other factors. Neuropsychopharmacology. (2020) 45:104–20. 10.1038/s41386-019-0478-5 31408876 PMC6879647

[17] Berry RB Brooks R Gamaldo C Harding SM Lloyd RM Quan SF. AASM scoring manual updates for 2017 (Version 2.4). J Clin Sleep Med. (2017) 13:665–6. 10.5664/jcsm.6576 28416048 PMC5406946

[18] Quan S Gillin JC Littner M Shepard J. Sleep-related breathing disorders in adults: recommendations for syndrome definition and measurement techniques in clinical research. editorials. Sleep. (1999) 22:662–89. 10.1093/sleep/22.5.667 10450601

[19] Chiu MJ Yang SY Horng HE Yang CC Chen TF Chieh JJ. Combined plasma biomarkers for diagnosing mild cognition impairment and Alzheimer's disease. ACS Chem Neurosci. (2013) 4:1530–6. 10.1021/cn400129p 24090201 PMC3867966

[20] Chiu MJ Chen YF Chen TF Yang SY Yang FP Tseng TW. Plasma tau as a window to the brain-negative associations with brain volume and memory function in mild cognitive impairment and early Alzheimer's disease. Hum Brain Mapp. (2014) 35:3132–42. 10.1002/hbm.22390 24129926 PMC6869439

[21] Lue L-F Sabbagh MN Chiu M-J Jing N Snyder NL Schmitz C. Plasma levels of Aβ42 and tau identified probable Alzheimer's dementia: findings in two cohorts. Front Aging Neurosci. (2017) 9:226. 10.3389/fnagi.2017.00226 28790911 PMC5522888

[22] Jiao F Yi F Wang Y Zhang S Guo Y Du W. The validation of multifactor model of plasma Aβ 42 and total-Tau in combination with MoCA for diagnosing probable Alzheimer disease. Front Aging Neurosci. (2020) 12:212. 10.3389/fnagi.2020.00212 32792940 PMC7385244

[23] Liguori C Maestri M Spanetta M Placidi F Bonanni E Mercuri NB. Sleep-disordered breathing and the risk of Alzheimer's disease. Sleep Med Rev. (2021) 55:101375. 10.1016/j.smrv.2020.101375 33022476

[24] Liguori C Mercuri NB Izzi F Romigi A Cordella A Sancesario G. Obstructive sleep apnea is associated with early but possibly modifiable Alzheimer's disease biomarkers changes. Sleep. (2017) 40:zsx011. 10.1093/sleep/zsx011 28329084

[25] Dingli K Fietze I Assimakopoulos T Quispe-Bravo S Witt C Douglas NJ. Arousability in sleep apnoea/hypopnoea syndrome patients. Eur Respir J. (2002) 20:733–40. 10.1183/09031936.02.00262002 12358354

[26] Chen H-C Lin C-M Lee M-B Chou P. The relationship between pre-sleep arousal and spontaneous arousals from sleep in subjects referred for diagnostic polysomnograms. J Chin Med Assoc. (2011) 74:81–6. 10.1016/j.jcma.2011.01.016 21354085

[27] Yaffe K Laffan AM Harrison SL Redline S Spira AP Ensrud KE. Sleep-disordered breathing, hypoxia, and risk of mild cognitive impairment and dementia in older women. JAMA. (2011) 306:613–9. 10.1001/jama.2011.1115 21828324 PMC3600944

[28] Buratti L Viticchi G Falsetti L Cagnetti C Luzzi S Bartolini M. Vascular impairment in Alzheimer's disease: the role of obstructive sleep apnea. J Alzheimers Dis. (2014) 38:445–53. 10.3233/JAD-131046 23985418

[29] Scoring E. EEG arousals: scoring rules and examples: a preliminary report from the Sleep Disorders Atlas Task Force of the American Sleep Disorders Association. Sleep. (1992) 15:174–84. 10.1093/sleep/15.2.174 11032543

[30] Liguori C. Orexin and Alzheimer's disease. Behav Neurosci Orexin/Hypocretin. (2016) 33:305–22. 10.1007/7854_2016_50 28012089

[31] Vanderheyden WM Lim MM Musiek ES Gerstner JR. Alzheimer's disease and sleep-wake disturbances: amyloid, astrocytes, and animal models. J Neurosci. (2018) 38:2901–10. 10.1523/JNEUROSCI.1135-17.2017 29563238 PMC6596072

[32] Peter-Derex L Yammine P Bastuji H Croisile B. Sleep and Alzheimer's disease. Sleep Med Rev. (2015) 19:29–38. 10.1016/j.smrv.2014.03.007 24846773

[33] Ahnaou A Drinkenburg W. Sleep, neuronal hyperexcitability, inflammation and neurodegeneration: does early chronic short sleep trigger and is it the key to overcoming Alzheimer's disease? Neurosci Biobehav Rev. (2021) 129:157–79. 10.1016/j.neubiorev.2021.06.039 34214513

[34] Ju Y-ES Ooms SJ Sutphen C Macauley SL Zangrilli MA Jerome G. Slow wave sleep disruption increases cerebrospinal fluid amyloid-β levels. Brain. (2017) 140:2104–11. 10.1093/brain/awx148 28899014 PMC5790144

[35] Zhang X Le W. Pathological role of hypoxia in Alzheimer's disease. Exp Neurol. (2010) 223:299–303. 10.1016/j.expneurol.2009.07.033 19679125

[36] Smith MA Rottkamp CA Nunomura A Raina AK Perry G. Oxidative stress in Alzheimer's disease. Biochim Biophys Acta MolBasisDis. (2000) 1502:139–44. 10.1016/S0925-4439(00)00040-5 10899439

[37] Cordone S Annarumma L Rossini PM De Gennaro L. Sleep and β-amyloid deposition in Alzheimer disease: insights on mechanisms and possible innovative treatments. Front Pharmacol. (2019) 10:695. 10.3389/fphar.2019.00695 31281257 PMC6595048

[38] Irwin MR Vitiello MV. Implications of sleep disturbance and inflammation for Alzheimer's disease dementia. Lancet Neurol. (2019) 18:296–306. 10.1016/S1474-4422(18)30450-2 30661858

[39] Bubu OM Andrade AG Umasabor-Bubu OQ Hogan MM Turner AD de Leon MJ. Obstructive sleep apnea, cognition and Alzheimer's disease: a systematic review integrating three decades of multidisciplinary research. Sleep Med Rev. (2020) 50:101250. 10.1016/j.smrv.2019.101250 31881487 PMC7593825

[40] Shi Y Feng Y Chen X Ma L Cao Z Shang L. Serum neurofilament light reflects cognitive dysfunctions in children with obstructive sleep apnea. BMC Pediatr. (2022) 22:449. 10.1186/s12887-022-03514-9 35879699 PMC9316320

[41] Tsai C-Y Hsu W-H Lin Y-T Liu Y-S Lo K Lin S-Y. Associations among sleep-disordered breathing, arousal response, and risk of mild cognitive impairment in a northern Taiwan population. J Clin Sleep Med. (2022) 18:1003–12. 10.5664/jcsm.9786 34782066 PMC8974381

[42] Gillis C Mirzaei F Potashman M Ikram MA Maserejian N. The incidence of mild cognitive impairment: a systematic review and data synthesis. Alzheimers Dement Diagn Assess Dis Monit. (2019) 11:248–56. 10.1016/j.dadm.2019.01.004 30911599 PMC6416157

[43] Liu DS Pan XD Zhang J Shen H Collins NC Cole AM. APOE4 enhances age-dependent decline in cognitive function by down-regulating an NMDA receptor pathway in EFAD-Tg mice. Mol Neurodegener. (2015) 10:7. 10.1186/s13024-015-0002-2 25871877 PMC4391134

[44] Lee YF Gerashchenko D Timofeev I Bacskai BJ Kastanenka KV. Slow wave sleep is a promising intervention target for Alzheimer's disease. Frontiers Neurosci. (2020) 14:705. 10.3389/fnins.2020.00705 32714142 PMC7340158

[45] Ding L Chen B Dai Y Li Y. A meta-analysis of the first-night effect in healthy individuals for the full age spectrum. Sleep Med. (2022) 89:159–65. 10.1016/j.sleep.2021.12.007 34998093

