# Supplementary material for: Associations between risk of Alzheimer's disease and obstructive sleep apnea, intermittent hypoxia, and arousal responses: A pilot study
Source: Front Neurol. 2022 Nov 30;13:1038735. doi: 10.3389/fneur.2022.1038735 (PMC9747943; doi:10.3389/fneur.2022.1038735)
Supplement: Supplementary file 1 [file Data_Sheet_1.docx]

**Supplementary**

Table S1. Demographic characteristics of all recruited participants (*n = 36*).

| Variable | Total | Variable | Total |
| --- | --- | --- | --- |
| Age | 52.25 ± 11.17 | **SDB indices (events/h)** |  |
| Sex (male/female) |  | AHI | 40.57 ± 20.28 |
| BMI (kg/m^2^) | 29.06 ± 4.53 | AI | 33.41 ± 21.75 |
| Neck circumference (cm) | 40.11 ± 7.75 | AI_NREM_ | 12.39 ± 15.38 |
| Waist circumference (cm) | 96.04 ± 15.65 | AI_REM_ | 16.8 ± 17.17 |
| **Biomarker levels** |  | HI | 27.64 ± 14.69 |
| T-Tau (pg/mL) | 23.85 ± 5.13 | HI_NREM_ | 27.29 ± 15.11 |
| Aβ_42_ (pg/mL) | 16.41 ± 0.97 | HI_REM_ | 30.14 ± 20.74 |
| Aβ_42_ / T-Tau | 0.71 ± 0.12 | **Arousal indices (events/h)** |  |
| Aβ_42_ × T-Tau (pg/mL)^2^ | 394.9 ± 107.72 | ArI | 22.95 ± 13.91 |
| **OSA severity** |  | ArI_NREM_ | 22.75 ± 14.59 |
| Normal, *n* (%) | 1 (2.78%) | ArI_REM_ | 24.45 ± 15.34 |
| Mild, *n* (%) | 4 (11.11%) | Sp-ArI | 6.06 ± 4.7 |
| Moderate, *n* (%) | 4 (11.11%) | Sp-ArI_NREM_ | 6.13 ± 4.77 |
| Severe, *n* (%) | 27 (75%) | Sp-ArI_REM_ | 6.7 ± 7.93 |
| **Sleep architecture parameters** |  | R-ArI | 14.9 ± 12.88 |
| Sleep efficiency (%) | 78.35 ± 14.3 | R-ArI_NREM_ | 14.63 ± 13.65 |
| Wake (% of SPT) | 17.27 ± 12.75 | R-ArI_REM_ | 15.78 ± 14.91 |
| NREM (% of SPT) | 71.14 ± 10.88 | Sn-ArI | 0.36 ± 1.2 |
| REM (% of SPT) | 11.57 ± 6.2 | Sn-ArI_NREM_ | 0.36 ± 1.23 |
| WASO (min) | 58.84 ± 40.79 | Sn-ArI_EM_ | 0.32 ± 1.14 |
| TST (min) | 287.09 ± 52.73 | L-ArI | 1.64 ± 2.07 |
| **Oximetry parameters** |  | L-ArI_NREM_ | 1.62 ± 3.29 |
| Mean SpO_2_ (%) | 94.59 ± 1.95 | L-ArI_REM_ | 1.65 ± 2.03 |
| Minimum SpO_2_ (%) | 79.78 ± 8.73 |  |  |
| ODI-3% (events/h) | 36.16 ± 19.2 |  |  |
| Abbreviations: BMI: body mass index; OSA: obstructive sleep apnea; SPT: sleep period time; NREM: nonrapid eye movement; REM: rapid eye movement; WASO: wake time after sleep onset; TST: total sleep time; SpO_2_: peripheral arterial oxygen saturation (measured using pulse oximetry); ODI-3%*:* oxygen desaturation index ≥ 3%; SDB: sleep-disordered breathing; AHI: apnea–hypopnea index; AI: apnea index; AI_NREM_: apnea index in nonrapid eye movement stage; AI_REM_: apnea index in rapid eye movement stage; HI: hypopnea index; HI_NREM_: hypopnea index in nonrapid eye movement stage; HI_REM_: hypopnea index in rapid eye movement stage; ArI: arousal index; ArI_NREM_: arousal index in nonrapid eye movement stage; ArI_REM_: arousal index in rapid eye movement stage; Sp-ArI: spontaneous arousal index; Sp-ArI_NREM_: spontaneous arousal index in nonrapid eye movement stage; Sp-ArI_REM_: spontaneous arousal index in rapid eye movement stage; R-ArI: respiratory arousal index; R-ArI_NREM_: respiratory arousal index in nonrapid eye movement stage; R-ArI_REM_: respiratory arousal index in rapid eye movement stage; Sn-ArI: snore arousal index; Sn-ArI_NREM_: snore arousal index in nonrapid eye movement stage; Sn-ArI_REM_: snore arousal index in rapid eye movement stage; L-ArI: limb movement arousal index; L-ArI_NREM_: limb movement arousal index in nonrapid eye movement stage; L-ArI_REM_: limb movement arousal index in rapid eye movement stage. | | | |

Table S2. Odd ratios (ORs) associated with sleep-disordered breathing indices in the low- and high-risk groups (with 403.72 as the cutoff; low-risk group: *n=22;* high-risk group: *n=14*).

| Variable (events/h) | Crude OR (95% CI) ^a^ | Adjusted OR (95% CI) ^b^ |
| --- | --- | --- |
| Oximetry parameter |  |  |
| ODI-3% (events/h) | 1.07 (1.02 to 1.13) ** | 1.09 (1.02 to 1.15) ** |
| SDB index (events/h) |  |  |
| AHI | 1.07 (1.02 to 1.13) ** | 1.08 (1.02 to 1.15) ** |
| AI | 1.04 (0.99 to 1.1) | 1.04 (0.99 to 1.09) |
| AI_NREM_ | 1.04 (0.99 to 1.09) | 1.03 (0.99 to 1.08) |
| AI_REM_ | 1.02 (0.98 to 1.07) | 1.03 (0.98 to 1.07) |
| HI | 1.07 (1.01 to 1.14) * | 1.12 (1.03 to 1.22) * |
| HI_NREM_ | 1.08 (1.02 to 1.15) * | 1.12 (1.03 to 1.21) ** |
| HI_REM_ | 1.02 (0.99 to 1.06) | 1.04 (0.99 to 1.1) |
| Abbreviations: ODI-3%*:* oxygen desaturation index ≥ 3%; SDB: sleep-disordered breathing; AHI: apnea–hypopnea index; AI: apnea index; AI_NREM_: apnea index in nonrapid eye movement stage; AI_REM_: apnea index in rapid eye movement stage; HI: hypopnea index; HI_NREM_: hypopnea index in nonrapid eye movement stage; HI_REM_: hypopnea index in rapid eye movement stage.  ^a^ Simple logistic regression models.  ^b^ Multivariable logistic regression models were adjusted for age, sex, and body mass index.  **p* < 0.05; ***p* < 0.01. | | |

Table S3. Odd ratios associated with arousal indices in low- and high-risk groups (with 403.72 as the cutoff point; low-risk group: *n=22;* high-risk group: *n=14*).

| Variable (events/h) | Crude OR (95% CI) ^a^ | Adjusted OR (95% CI) ^b^ |
| --- | --- | --- |
| ArI | 1.06 (1.0 to 1.12) | 1.06 (1.0 to 1.13) |
| ArI_NREM_ | 1.06 (1.0 to 1.12) * | 1.06 (1.0 to 1.13) * |
| ArI_REM_ | 1.02 (0.97 to 1.06) | 1.01 (0.96 to 1.07) |
| Sp-ArI | 0.99 (0.93 to 1.06) | 1.01 (0.94 to 1.09) |
| Sp-ArI_NREM_ | 0.99 (0.93 to 1.05) | 1.01 (0.94 to 1.08) |
| Sp-ArI_REM_ | 0.97 (0.9 to 1.04) | 0.98 (0.91 to 1.06) |
| R-ArI | 1.13 (1.02 to 1.26) * | 1.14 (1.01 to 1.28) * |
| R-ArI_NREM_ | 1.12 (1.02 to 1.23) * | 1.12 (1.01 to 1.25) * |
| R-ArI_REM_ | 1.02 (0.99 to 1.05) | 1.02 (0.98 to 1.06) |
| Abbreviations: CI: confidence interval; ArI: arousal index; ArI_NREM_: arousal index in nonrapid eye movement stage; ArI_REM_: arousal index in rapid eye movement stage; Sp-ArI: spontaneous arousal index; Sp-ArI_NREM_: spontaneous arousal index in nonrapid eye movement stage; Sp-ArI_REM_: spontaneous arousal index in rapid eye movement stage; R-ArI: respiratory arousal index; R-ArI_NREM_: respiratory arousal index in nonrapid eye movement stage; R-ArI_REM_: respiratory arousal index in rapid eye movement stage.  ^a^ Simple logistic regression models.  ^b^ Multivariable logistic regression models were adjusted for age, sex, and body mass index.  **p* < 0.05; ***p* < 0.01. | | |
